# Adolescents’ perspectives on a novel digital treatment targeting eating disorders: a qualitative study

**DOI:** 10.1186/s12888-024-05866-1

**Published:** 2024-06-05

**Authors:** Guri Holgersen, Sara Elisabeth Abdi-Dezfuli, Solveig Friis Darrud, Ester Marie Stornes Espeset, Irene Bircow Elgen, Tine Nordgreen

**Affiliations:** 1 Division of Psychiatry, Haukeland University Hospital, Post OfficeBox 1400, Bergen, N-5021 Norway; 2 Department of Global Public Health and Primary Care, University of Bergen, Bergen, Norway; 3 Faculty of Psychology, University of Bergen, Bergen, Norway; 4 Department of Child and Adolescent Psychiatry, Helse Fonna, Haugesund, Norway; 5 Department of Child and Adolescent Psychiatry, Haukeland University Hospital, Bergen, Norway; 6 Department of Clinical Medicine, University of Bergen, Bergen, Norway

**Keywords:** Eating disorders, Adolescents, Digital treatment, Qualitative study, Treatment development

## Abstract

**Background:**

Eating disorders in adolescence are associated with high psychological distress, impaired function and high comorbidity. Despite the severity, eating disorders remain highly underdiagnosed and untreated. Digital technology provides promising opportunities for treatment, however studies focusing on digital treatments for adolescents with eating disorders are lacking. The main aim of this study was to explore the perspectives of adolescents with lived experience of eating disorders on factors they deemed to be relevant in the development of a novel digital treatment.

**Methods:**

A qualitative intervention development study using semi-structured individual interviews. Data collection, coding and analysis were conducted using the principles of reflexive thematic analysis. Participants were adolescents aged 16–19 years, with a self-reported diagnosis of anorexia nervosa, bulimia nervosa or binge eating disorder, currently in the final phase or completed psychological treatment for an eating disorder within the last five years.

**Results:**

A total of 16 adolescents participated in the study, all females. Mean age was 17 ½ years (SD = 1.01). An in-depth understanding of the adolescents’ perspectives was developed into three themes: Facilitating self-awareness and readiness to change; Strengthening interpersonal relationships and decreasing social isolation; Ensuring feeling seen and motivating regular use.

**Conclusions:**

This study provides a unique insight into the perspectives of adolescents with lived experience of eating disorders. The uptake and engagement can be optimized in a novel digital treatment for eating disorders by taking the adolescents perspectives into consideration.

**Supplementary Information:**

The online version contains supplementary material available at 10.1186/s12888-024-05866-1.

## Background

Eating disorders in adolescence are associated with high psychological distress and social costs for the adolescents and their families [1–4]. In addition, eating disorders disrupt psychosocial functioning [5–7], impair physical health [4, 8], and are associated with high psychiatric comorbidity (> 70%) [9]. The lifetime prevalence of all DSM-5 eating disorders [6] among adolescent and young adult women ranges from 5.5 to 17.9%, and from 0.6 to 2.4% among young men [10]. Additionally, the COVID-19 pandemic has contributed to a significant increase in the number of adolescents diagnosed with an eating disorder [10, 11]. Several evidence-based treatments are available; especially family-based treatment (FBT) and individual eating-disorder-focused cognitive behavioural therapy (CBT-ED) have proven effective for adolescents [12].

Despite the severity and negative impact, eating disorders remain highly underdiagnosed and untreated [1, 13]. Treatment-seeking is especially low among adolescents [14–16] with only 10–20% of those in need seeking treatment [14–16]. Barriers to help-seeking behaviour are feelings of shame and fear of stigma from families, friends, and health professionals [13, 17, 18]. Other barriers are denial or a lack of awareness of the severity of their eating disorder [17, 19]. Among those who access treatment there is a high dropout and relapse rate [20–22].

Combining evidence-based treatment with technology might address some of these challenges [23–26]. Technology-enhanced interventions (i.e., intervention delivered via computers, smartphones, or other digital means) for eating disorders might reduce barriers to help seeking, reduce symptoms and prevent relapse [27, 28]. Internet-delivered CBT-ED and FBT appear to be promising approaches [24–27], but studies involving adolescents are limited [24, 25, 29] and dropout continues to be a major issue [24, 26, 30]. Consequently, there is a need to develop engaging digital mental health interventions for adolescents with eating disorders [25, 29].

A potential solution to improve engagement and adherence in digital treatments is to involve the users when developing the interventions [31–33]. In developing effective behaviour change digital treatments, one needs to facilitate a profound understanding of the perspective and psychosocial context of the people who will use them [34, 35]. It is important to understand what the users want, how technology can fit into their lives and how the digital treatment can solve a problem or fill a gap to meet their needs [31, 34]. Development of technologies for adolescent mental health requires a particular understanding of their needs and preferences [35, 36]. It is essential to match the digital treatment with the adolescents’ range of interests, their goals and ensure developmental and age-appropriate considerations [35, 36]. Additionally, the technology needs to include the right age-tailored functionalities and delivery options to enhance the users’ engagement [32, 35].

There is a range of approaches, research methodologies, and frameworks to ensure involvement of users in developing technology-enhanced interventions [34, 37–39]. The person-based approach is an evidence-based method for developing user-friendly and effective behaviour change treatments [34]. The approach provides guidance on how to integrate evidence-based treatments with the users’ needs and perspectives when developing a novel treatment [34]. Existing theory will offer insight into effective behaviour change techniques to consider, but there is often no clear evidence on which are most important or how best to implement them in a particular context [34]. The person-based approach utilizes qualitative research to explore and analyse the attitudes, needs, and situations of the intervention’s intended users [34]. The aim is to select intervention components that are most acceptable, feasible, and salient to them, thereby ensuring that the digital treatment is engaging, persuasive and effectively change behaviour [34]. The qualitative interviews, along with the existing evidence-base, are guiding principles of designing the intervention [34].

In this study, we utilized a person-based approach that explores adolescents with lived experience of eating disorders’ perspectives in order to develop a novel digital treatment for adolescents with eating disorders. The main aim of this study was to explore the perspectives of adolescents with lived experience of eating disorders on factors they deemed to be relevant in the development of a novel digital treatment.

## Method

### Design

We conducted a qualitative study using semi-structured individual interviews. The interview guide [see Additional file 1] included closed and open-ended questions and was designed to ensure that we obtained an in-depth understanding of the adolescents’ experiences, needs and interests [40]. Reflexive thematic analysis was used to identify patterns of meaning across the qualitative interviews [41]. The methodological criteria were checked retrospectively according to the Consolidated criteria for reporting qualitative research checklist [see Additional file 2] [42].

### Participants and procedures

We aimed to include a sample of adolescents with lived experience of eating disorders, reflecting the age and target group for the transdiagnostic novel digital treatment. We used convenience sampling method to enrol participants. The following inclusion criteria were applied: (a) A self-reported diagnosis of anorexia nervosa, bulimia nervosa or/and binge eating disorder [6, 7], (b) currently in the final phase or completed treatment for an eating disorder within the last five years, (c) age between 16 and 19 years. Exclusion criteria were (a) Self-reported ongoing substance abuse, manic or/and psychotic episode, (b) self-reported autism spectrum disorder, (c) currently receiving inpatient treatment for eating disorder.

Information about the study was provided at the waiting areas in outpatient clinics, at high schools and at user organizations. Due to slow recruitment, we added posters in social media. Interested participants were provided with a link to the study website for information about the study and digital screening assessment. Eligible participants were contacted by telephone to validate the inclusion and exclusion criteria. The semi-structured interviews were conducted per phone from mid-December 2022 until mid-January 2023. The mean interview duration was 34 min. All interviews were audio recorded and transcribed.

### Data analysis

We used reflexive thematic analysis to capture and explore the participants own perspectives and understandings [41]. A relativist constructionist theoretical framework was chosen, aiming to interrogate and unpack the realities that are expressed within the dataset [41]. We chose an inductive approach using the dataset as a starting point for engaging with meaning, and codes and themes were developed aiming to explore the participants experiences and perspectives [41]. We followed Braun and Clark’s six-phase method to describe how patterns of meanings were combined into broader conceptualizations:

First, we immersed ourselves in the transcript material to gain a deeper understanding of the content [41]. Secondly, a systematic line-by-line data coding was conducted, tagging all segments of the text what was potentially relevant to our research question [41]. All transcripts were coded by the first author (GH). The codes were a combination of semantic and latent codes. Throughout the dataset we had an emphasis on latent codes focusing on a deeper and conceptual level of meaning (e.g., reduce shame), while semantic codes were chosen when the participants answers were close to the overt meaning (e.g., difficulties with friends). In phase three, the codes were grouped into initial themes based on our aim of the study [41]. Further, the initial themes were checked against the coded data as well as the entire dataset. We focused on themes reflecting a central, organizing concept that could be related to the dataset, research question, and a broader context [41]. In phase five, we developed the themes further as we engaged in a more fine-tuned analytical work. We wrote definitions and developed informative and concise names for each theme [41]. In the final stage, we wrote the analytic report, giving us a final opportunity to refine the analysis [41].

The aim of our study, the sample specificity, the use of established theory, quality of dialogue, and the analysis strategy indicate that the sample held sufficient information power to develop new knowledge [43].

### Reflexivity

In approaching this research, the first author (GH) was aware that her personal reflexivity, position as a PhD candidate (functional reflexivity) and clinical experience would influence the collecting, analysing and interpretating of the data. Throughout the research process a reflexive research journal was kept to record feelings and assumptions, and how participants’ accounts fitted with clinical knowledge and experience [41]. One example of this reflexive process was when the first author was pondering about whether the latent code “difficulties with change” were based on the interviews themselves or also affected by previous clinical experience. The authors analysing the data (GH, SEAD, SEAD, TN) reflected with members of the research team, with colleagues, and peers to ensure different perspectives, discover biases or assumptions and to get a more nuanced analysis. The first author (GH) moved between phases, regularly returning to the original transcripts to ensure that emerging themes were grounded in the data. All decisions made during the analysis and rationale of these were documented in a qualitative data analysis tool (NVivo 13), this to increase validity [44] and ensure qualitative sensibility [41].

## Results

**Fig. 1:**
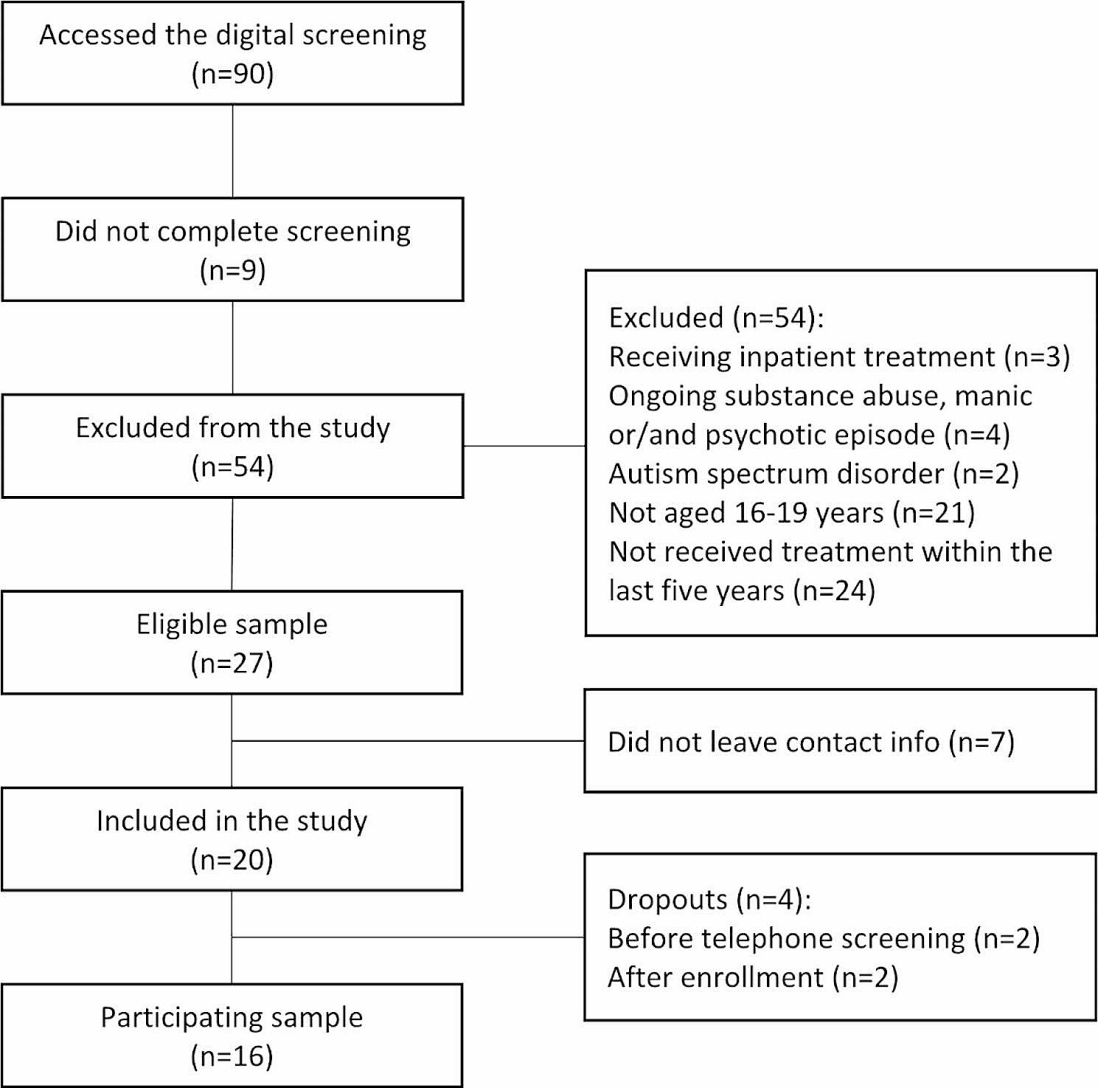
Flowchart of participants approximately

A total of 90 participants accessed the digital screening portal (Fig. 1). Of these, 54 did not meet the criteria for participation and 9 did not complete the online screening. Of the 27 suitable participants remaining, 20 left their contact information and were invited to participate in the study and signed informed consent. Four participant dropped-out before the interviews and 16 adolescents (80%) participated in the study. The mean age was 17.4 years (SD = 1.01, range 16–19), all participants were female.

In line with the procedure, no clinical characteristics data were gathered. As anticipated, none of the participants had prior experience with digital treatment. All the participants were elaborating their answers with personal experiences regarding having an eating disorder and receiving face-to-face treatment. We gained an in depth understanding of the specificity of the experiences and knowledge among the participants.

The participants perspectives were developed into three main themes, with associated subthemes (Table 1). The following excerpts have been selected since they were found to be representative of the data, illustrating the essence in the identified subthemes.

**Table 1:** Themes and subthemes of adolescents’ perspectives on a novel digital treatment targeting eating disorders

Themes	Subthemes
Facilitating self-awareness and readiness to change	Knowledge of disorder Perceptions of control Management of emotions
Strengthening interpersonal relationship and decreasing social isolation	Openness about own disorder Managing social situations Parental understanding
Ensuring feeling seen and motivating regular use	Tailored content Engaging features Treatment accessibility

### Facilitating self-awareness and readiness to change

This theme was developed from the adolescents’ experiences regarding how learning about various aspects of their eating disorder contributed to increased self-awareness and influenced their motivation for treatment. To facilitate self-awareness and readiness to change in a digital treatment, adolescents benefit from understanding the disorder, changing their perception of control, and managing their emotions.

#### Knowledge of disorder

The adolescents expressed how it is normal to seek out information from social media, making it difficult to navigate in terms of what information could be considered reliable or not. At some point, the adolescents found it difficult to distinguish between fact-based knowledge and misinformation about the disorder: “*For me, the internet is super scary, because a lot of the information given is incorrect and can be misinterpreted”* (ID13). The adolescents highlighted that they would consider a digital treatment created by the hospital to contain trustworthy information and involving them in the development process would make the intervention extra reliable.

The adolescents’ voiced how they, during treatment, realized that they had been exposed to a lot of misleading information about eating disorders, and that there were also a lot of information they did not obtain. Particularly, knowledge regarding the impact and consequences eating disorders has on physical and mental health, was lacking. Thus, information about the side-effects of an eating disorder was highlighted as an important factor to be included in the digital treatment.

*“It gets harder at school. It’s very difficult to concentrate if you haven’t eaten. Many people have such long-term effects on their memory. It is important to point out that it [the eating disorder] has stupid consequences”* (ID9).

Additionally, the adolescents voiced that increased knowledge made them realize that their disorder prevented them for achieving their goals. For several adolescents this was linked to motivation and compliance of treatment: *«Knowledge about the eating disorder helped me find motivation to continue with the treatment, because it was a bit tough. Knowing about the side effects and how it can affect the future and what you can do with it yourself, was important»* (ID6).

#### Perceptions of control

All adolescents highlighted how important it was during their illness trajectories to achieve and maintain a sense of control over different areas in life. Achieving a sense of control gave them a feeling of accomplishment, increased self-confidence, strengthened their self-esteem and was a tool for tolerating distress. Behavior used to achieve and maintain control could for example be starvation: *“You feel better when you have a very low weight”* (ID5). Experiences from treatment were that awareness of this disordered behaviour made them realise that their perceptions of control and their actions were leaving them with a self-critical inner voice that resulted in negative emotions and maintained a low-self-esteem.

*“You lose control of yourself. You don’t think about what you’re doing when you’re in the middle of it, until you look back and realise that this wasn’t healthy at all. So, it’s very easy to lose control and think you’re in control, but then you’re not”* (ID13).

Realising that their behavioural patterns were maintaining a low-self-esteem did not necessarily indicate readiness to change. Several adolescents voiced that gaining weight amplified an internal conflict because they felt better when they had a low weight. However, readiness to change was linked to changing their sense of control to something more sustainable, instead of focusing on removing it: *“In my case, it was to shift the sense of mastery I got from controlling things with food to something else, which was healthier than starving myself*” (ID12).

#### Management of emotions

The adolescents described significant difficulties in understanding, enduring, and manging emotions during their illness trajectories. However, the connection between disorder and emotions varied between them; some experienced these difficulties because of the disorder: *“When you are malnourished you often have anxiety and depressive symptoms, and I think it is very important to learn methods and ways to cope with these”* (ID6). While other experienced these difficulties as a triggering factor: “*You control your emotions by binge eating. Food becomes a tool to control your emotions*” (ID4).

The ability to endure emotional distress was especially emphasised as a challenge. The adolescents described how they tended to become overwhelmed by stressful situations and turned to unhealthy and destructive ways of coping with their emotions: *“If something negative happened I wouldn’t eat*” (ID14). Further, tolerating uncertainty was described as extremely difficult to manage when the adolescents were asked to reduce, or let go of, their unhealthy control during treatment: *“When you start a journey to recovery, whether it’s voluntary or involuntary, it’s very scary. Because you are used to being in control of everything. If you eat that, you must do so, or you cannot eat that. You have a lot of control. Letting go of it triggers a sense of panic. You don’t quite know what you’re doing. So, I think it’s a good thing to get help managing that feeling of losing control”* (ID10).

Managing emotional distress was not only related to challenges directly associated with eating disorders, but also difficulties occurring in life because of being ill (e.g., difficulties with friendships, conflict with parents). The adolescents also highlighted that emphasising on managing difficult emotions was important throughout treatment. One adolescent put it like this: “*Dealing with difficult emotions is important to focus on throughout treatment. After all, you must cope with both starting to eat again and maintaining eating. So that you don’t go back to old patterns as soon as you weigh enough. You must also cope being a normal weight again after an eating disorder”* (ID5).

### Strengthening interpersonal relationship and decreasing social isolation

This theme was developed from the adolescents’ experiences with difficulties in interpersonal relationships. To strengthen interpersonal relationships and decrease social isolation in a digital treatment, adolescents benefit from being open about their own disorder, manging social situations, and have parents who understand them.

#### Openness about own disorder

The adolescents described how experiencing fear of stigma caused them restraining their communication regarding their eating disorder: *“I feel that eating disorders are often thoughts that each individual carry with them alone. It is quite taboo to talk to others about this. Many says nothing to others”* (ID4).

The adolescents particularly highlighted their experiences with how reduced openness about their own disorder created difficulties in maintaining friendships: *“Some have friends who understand them or have been in the same situation. But not everyone feels they can talk to their friends, and not everyone likes to do the same things they did without these difficulties”* (ID2).

The adolescents expressed how difficulties with being open about their disorder led to them spending a lot of energy to “act normal” among friends. One adolescent put it like this: *“It can often be very difficult around friends. You don’t always want to say everything. You try to be as normal as possible without them noticing that something is wrong”* (ID1). The fear of peers paying attention to them also created problems for the adolescents at school: *“It is difficult with classmates. How to deal with the fact that you must leave earlier because you are going to the doctor, or that you must sit inside in the break to eat*” (ID15).

According to the adolescents, being open about own disorder can help maintain friendships: “*I think it’s important for friends to know that: “Yes, right now I’m struggling with an eating disorder but I’ll try my best to be with you sometimes, so you know I’m still here when I get well enough so that we can be together in a normal way again”* (ID5).

#### Managing social situations

The adolescents described how the eating disorder became all-consuming and thus caused them to lose friends: *“You disappear from your friends when you have an eating disorder. You lose a lot of friends when you are very ill*” (ID16). Reduced social participation was also linked to anxiety: *“I had anxiety symptoms due to the eating disorder. Especially anxiety about food and social settings”* (ID3).

These factors increased a sense of loneliness and social isolation: “*You become very lonely when you have an eating disorder*” (ID3). Another adolescent put it like this: “*There was a lot of social situations I didn’t dare to be a part of, because of the eating disorder. I lost a lot of my social life. You must not isolate yourself. You* *should try to participate in activities. Eating with others; Yes, it’s difficult, but it’s not dangerous*” (ID9). Eating with others was namely one of the biggest social challenges throughout the adolescents’ illness trajectory: “*In many social settings it is food that brings people together. It can lead to not joining in on social events. Because the fear of food, that you must eat*” (ID13).

The adolescents highlighted the importance of focusing on friendship and social participation throughout the treatment: *“In a way it becomes two challenges. First you must recover from the eating disorder and then you must get back to normal, which has suddenly become very scary. To prevent that from happening you can perhaps have some focus on it [friendships] throughout. Even if you don’t eat together there are many other things you can do together*” (ID7).

#### Parental understanding

According to the adolescents the eating disorders enhanced feelings of shame and guilt in the face of family: *“I remember feeling very embarrassed in front of my family. I can still feel a bit guilty, in a way”* (ID14). The eating disorders led to communication difficulties and affected the family dynamic: *“It [the eating disorder] affects the family a lot. Because in a sense they are with you in it. Being able to have a normal relationship can then be very difficult. Many conflicts can arise”* (ID1).

Increasing knowledge of eating disorders and guide parents on how to support their children could, according to the adolescents, reduce communication difficulties. One adolescent explained it like this: “*Parents or grandparents can be like: But you’re so pretty, why don’t you eat…Conflicts may arise in the family…Parents should receive information and tips on how they can help their children if they struggle with eating disorders*” (ID15).

Further, the adolescents emphasised the impotence of targeting distressing emotions when increasing awareness among parents. The adolescents voiced how they felt responsible for handling family members distressing emotions. In addition to dealing with their own illness, this became overwhelming: *“It becomes a lot of responsibility for the person who is ill to keep the family positive*” (ID7). One adolescent suggested that talking about shame and guilt could increase knowledge and awareness: *“I think it is very important to talk about feeling shame and guilt. Parents must work on not getting angry and blaming the person who is ill, while the person who is ill must work on not feeling so much guilt”* (ID5).

### Ensuring feeling seen and motivating regular use

This theme was developed from the adolescents’ experiences with face-to-face treatment. To ensure feeling seen and motivated in a digital treatment, adolescents benefit from tailored content, engaging features, and treatment accessibility.

#### Tailored content

The adolescents highlighted how different aspects of an eating disorder may not be universally experienced among those affected: *“There is great variation in what is the main problem for each individual”* (ID1). Not being a heterogenous group also applied for treatment efficacy; Several adolescents expressed how previous treatment encounters made them feel alienated by being exposed to therapeutic approaches that did not resonate with their subjective experiences. Additionally, this could elicit difficult emotions, including feelings of inadequacy or a sense of not being unwell enough to warrant treatment: *“It is quite typical that individuals who struggle with eating disorders tend to feel that they are not sick enough, or that they do not have it as tough as others do, or that they may not be underweight enough to deserve treatment”* (ID11).

Thus, the adolescents highlighted the importance of a digital treatment format that offers the user opportunities to determine their own direction: *“Eating disorder treatment can often put everyone in the same box, but it is quite different from person to person. I think there should be more individualisation, being able to choose one’s own directions and choose things that concern oneself*” (ID3). One adolescent suggested how the digital treatment could be tailored using different categories: *“If you struggle with a specific problem one day you can go to a category that contains that problem and then you can get tips or watch a video”* (ID9).

Further, the adolescents described how the content in the digital treatment should be tailored to the stage of the disorder that the user is experiencing: “*There are several types of eating disorders and different types of levels. So being able to know what the person needs some extra help with is important”* (ID10). Several adolescents suggested that mapping could be the solution to tailoring the content.

#### Engaging features

The adolescents described undergoing face-to-face treatment as challenging. Thus, having a content that motivate use was described as important in a digital treatment: *“First and foremost, it [the digital treatment] should contain motivational things. So that you feel a little more uplifted and not oppressed and sick when opening the APP. That you think; I will get well here”* (ID10).

Further, the adolescents described how an excessive focus on triggering topics such as food, calories, eating, and weight reduced their motivation for treatment, as they could induce difficult emotions like fear of stigma, guilt, and shame. The adolescents thus highlighted the importance of having a more positive focus when including potentially triggering topics in the digital treatment: “*Learn the positive things about food. Instead of thinking about calories, you think about the quality of food”* (ID12).

Another coherent engaging feature highlighted by the adolescents was motivational phrases: *“It would be a really good idea to have a place where you can get motivational words. Maybe phrases or quotes that can give you some strength back”* (ID9). Some adolescents suggested that a digital treatment should utilize motivational notifications, as this could increase the user’s uptake to the digital intervention and motivate them to enter it: *“Positive things, comments like; “You have to remember to eat”. Maybe positive notifications that you can get during the day”* (ID2).

#### Treatment accessibility

Face-to-face treatment was described as time-consuming and hard to combine with everyday life as an adolescent. Consequently, the adolescents highlighted that a digital treatment could be accessible and adapted to everyday life. A central element was that a digital treatment could increase school attendance and make life easier: *“It [a digital treatment] makes treatment more accessible, easier to adjust to your everyday life. If you’re in school, you do not have to leave school. I can imagine that it’s easier for some people to take part in treatment. Going to a therapist is very taboo, and many people feel like they must hide it”* (ID6). Several adolescents highlighted this latter fact, that a digital treatment might have a motivational factor for those who would not seek help in person: *“Having access to a digital treatment can be a huge help for many people, when they do not want to reach out to others or seek other help”* (ID5).

Further, the adolescents highlighted how a digital treatment also meant increased access to treatment compared to face-to-face treatment: *“When you have an eating disorder you may have treatment once or twice a week, and the rest of the time you are on your own. If you have a hard time, you can use it [the digital treatment], it is more accessible. And if you’re thinking about something or don’t dare book an urgent appointment, you can get more treatment there*” (ID3). Finally, the adolescents emphasised the importance of accessing guidance from a therapist in the digital treatment. They emphasised how the inclusion of a therapist could help increase the motivation for using the digital treatment: *“To be properly inspired you need a real person who accompanies you in the process*” (ID14).

## Discussion

The main aim of this study was to explore the perspectives of adolescents with lived experience of eating disorders on factors they deemed to be relevant in the development of a novel digital treatment. During the interviews, we gained an in-depth understanding of the adolescents’ experiences, needs and interests. The perspectives deemed to be relevant in the development of a novel digital treatment were developed into three themes: Facilitating self-awareness and readiness to change; Strengthening interpersonal relationship and decreasing social isolation; Ensuring feeling seen and motivating regular use.

### Facilitating self-awareness and readiness to change

The adolescents had gained misleading knowledge about their eating disorder through the Internet, something they had realized when they received face-to-face treatment. Searching for health-related information via the internet is common among adolescents in general [45], i.e., in Norway four out of five adolescents have used the internet to search for health-related information [46]. However, the quality of online information about eating disorders is of varying or poor quality [45, 47], resulting in misleading and potentially harmful knowledge about their own health [48, 49].

The adolescents had experienced an increased understanding of the nature of their psychopathology and subsequently themselves during face-to-face treatment. This is in accordance with the fact that psychoeducation is an essential part of psychological treatment for eating disorders [12, 50, 51]. The main aim of psychoeducation is to help the patients understand the nature and impact of eating-disordered behaviours and enable them to evaluate their relationship with the eating disorder [52].

Increased self-awareness made the adolescents realise how their eating disorder amplified intrapersonal conflicts and prevented them for achieving their goals. This realisation changed their identification with the eating disorders and improved readiness to change. This aligns with the meaning of self-awareness; the ability to focus on yourself and how your actions, thoughts, or emotions do or do not align with your internal standards [53, 54]. The present study indicates that increased self-awareness can function as a mediator for readiness to change for adolescents with eating disorders. The results emphasise the importance of facilitating self-awareness in treatment, whether digital or face-to-face, since low motivation to change is as a strong predictor of dropout among individuals struggling with an eating disorder [20, 21].

### Strengthening interpersonal relationship and decreasing social isolation

The reflexive analytic process implies that the adolescents had experienced challenges in their interpersonal relationships, a common clinical feature in the maintenance of eating disorders [55]. The adolescents described difficulties with friendships due to the eating disorder. This is in line with prior research reporting that adolescents with eating disorders are experiencing more interpersonal difficulties with friends, such as conflicts, communication difficulties, and alienation, compared to non-clinical samples [56, 57]. Adolescence is a period characterised by transitions of social relations away from family and towards peers [58], one relies more on peers for information and support [59]. Difficulties in managing these peer relations may, therefore, be of importance in the treatment of eating disorders, and according to the adolescents in the present study, being open about one’s own disorder is essential.

Further, the adolescents were avoiding situations where there was potential for social eating. The result from the present study highlights the importance of targeting management of social situation when development of a novel digital treatment for eating disorders. The findings align with previous research implicating that eating disorder treatment should not only focus on fear of eating and fear of gaining weight, but also fear related to social settings involving food [60, 61].

In addition, the adolescents had challenges in relationship to their parents. This is also reported from previous studies [3, 56, 57]. The family function plays an important role in the maintenance of eating disorders [3]. More specifically the adolescents highlighted feelings of shame and guilt in the face of family due to the eating disorder. Several studies show an association between lack of parental emotional acceptance and maintenance of eating disorder pathology [3]. Family-based treatment is recommended for adolescents with eating disorders [12, 62], however the treatment does not work for all families [63, 64]. Based on the experiences of the adolescents in the present study, management of emotions should also include parents in eating disorder treatments. This is supported by results from studies combing Dialectical behaviour therapy (focusing on managing and regulating emotions) with family-based treatment approaches [65, 66].

### Ensuring feeling seen and motivating regular use

The adolescents viewed eating disorders as highly heterogenous in terms of symptom presentation and treatment response. However, a common experience was being treated as one group which elicited feelings of inadequacy or a sense of not being ill enough to warrant treatment. Since, recommended treatments for eating disorders often are based on average symptom presentation [67–69] lack of personalisation might be the reason why these treatments do not work for a substantial percentage of individuals [26, 67]. Results from the present study address the importance of personalisation to meet the adolescents’ individual needs. Personalisation of treatment is viewed positively by individuals with eating disorders [70–72] and is especially highlighted as important for adolescents [73].

Further, the adolescents described how a digital treatment may give access to treatment at any time, and thus the ability to incorporate the treatment into their everyday lives. Accessibility has been suggested to influence engagement with digital treatments over time [74, 75]. The adolescents also linked treatment accessibility to the need for access to a therapist in a digital treatment. Knowing that a therapist can track their progress might increase motivation for reporting truthfully and increase engagement. The positive effects of having a therapist involved in digital treatment are supported by previous studies [76, 77]. However, for some participants it led to avoidance due to feeling shame when not following the treatment as planned [76]. These studies imply that individuals suffering from an eating disorder may have different needs regarding the involvement of therapists in a digital treatment. This underlines the importance of having the ability to individualise content to the specific person as this may increase acceptance and engagement with the digital treatment by making the adolescents feel seen and understood.

### Limitations

This study has some limitations that needs to be addressed. First, the current study used a convenience approach to recruitment. Since no clinical characteristics data were gathered, we cannot say with certainty that we reached a sample reflecting target group for the transdiagnostic novel digital treatment. The impression from the interviews was that the adolescents represented a variety, with the largest group having experiences with anorexia nervosa. Further, there were no male adolescents represented in the sample which may reflect the differences in prevalence [10]. Despite the sample limitation, it is our assessment that based on the current sample, aim of the study, the use of established theory, quality of dialogue, and the analysis strategy our sample held sufficient information power to develop new knowledge [43].

Secondly, all interviews were conducted by telephone. This can potentially limit the information obtained due to loss of nonverbal cues such as facial expressions and body language. This limitation was addressed in the interview and the adolescents were encouraged to speak up if any misunderstandings occurred during the interviews [see Additional file 1]. Nevertheless, telephone interviews are considered a good method for data collection if the goal of the study is to obtain information about participants life experience [78].

Lastly, an inductive approach was used to our data material meaning that the data formed the basis of our analytic process without attempting to fit it into pre-existing theories or frameworks [41]. One can never be completely detached from theoretical frameworks, professional interests, and our own perspectives on a subject and consequently, our analytical process was to some degree influenced by a deductive approach where our previous experiences, perspectives and knowledge might have influenced our analysis.

### Clinical implications

Technology-enhanced interventions for eating disorders needs to be designed to fit into the daily lives of those who will use them [31]. Ensuring that the intervention is relevant to the users and meet their needs will potentially improve engagement and clinical impact [31, 34]. The qualitative interviews in the current study gave the adolescents an opportunity to highlight what is important to them [79] and show how qualitative interviews can explore the needs and situations of the intervention’s intended users [34]. As a result, this study provides a unique insight into the perspectives of adolescents with lived experience of eating disorders and face-to-face treatment. We gained an in-depth understanding of important context-specific behavioural needs, problems, and challenges for adolescents struggling with an eating disorder before designing an intervention. Increasing adolescents’ self-awareness can significantly enhance their readiness to change, and feeling seen can positively influence their compliance. These implications are important given the low engagement and high dropout rates associated with eating disorders. Further, findings from the present study underline the importance of focusing on management of emotions related to intra- and interpersonal difficulties in treatment of eating disorders [80–82]. Additional, personalisation of treatment is essential to meet the adolescents’ individual needs. These understandings will help us to select intervention components that are acceptable, feasible, and salient for adolescents with eating disorders.

However, there are several factors negatively impacting adolescents’ engagement and interaction with technology-enhanced interventions e.g., lack of time, technical issues, concerns about privacy and anonymity and doubt regarding programme effectiveness [36]. We cannot say for certain that implementing the perspectives of the adolescents when creating the intervention will enhance engagement with the digital treatment. Additional, adolescents’ adherence to any medical treatment is challenging due to their developmental stage, emotional issues, and family dysfunction [83]. However, lack of adherence is often linked to applications not aligning with the preferences and goals of the people who will use the intervention [31, 36, 84]. This study contributes to novel understanding about the perspectives related to digital and non-digital treatment for adolescents with eating disorders. By taking the adolescents perspectives into consideration when developing a digital treatment for eating disorder, uptake and engagement can be optimised which in turn may reduce eating disorder symptoms.

## Conclusion

This study provides a unique insight into the perspectives of adolescents with lived experience of eating disorders. The uptake and engagement can be optimized in a novel digital treatment for eating disorders by taking the adolescents perspectives into consideration.

### Electronic supplementary material

Below is the link to the electronic supplementary material.

Supplementary Material 1: Additional file 1 (docx). Title of data: INTERVIEW GUIDE: Telephone interview. Description of data: The interview guide used in the semi-structured individual interviews

Supplementary Material 2: Additional file 2 (docx). Title of data: Consolidated criteria for reporting qualitative research (COREQ). Description of data: Reporting guidance for qualitative research

## Data Availability

Data generated, analysed, and reported during the current study are not publicly available due to it being potentially identifying, but are available in a slightly shortened, de-identified form from the corresponding author on reasonable request.
