# Supplementary material for: Adolescents’ perspectives on a novel digital treatment targeting eating disorders: a qualitative study
Source: BMC Psychiatry. 2024 Jun 5;24:423. doi: 10.1186/s12888-024-05866-1 (PMC11155031; doi:10.1186/s12888-024-05866-1)
Supplement: Supplementary file 1 — Supplementary Material 1: Additional file 1 (docx). Title of data: INTERVIEW GUIDE: Telephone interview. Description of data: The interview guide used in the semi-structured individual interviews [file 12888_2024_5866_MOESM1_ESM.pdf]

ID __

INTERVIEW GUIDE: Telephone interview

Information

"Hi, am I talking to xxx? My name is xx and I am calling as agreed to conduct a telephone interview. Is it still a good time to have the interview now? Great. Are you a somewhere you can talk, or do you need to go somewhere else?

Firstly, I just want to say that it's great that you can help with this project. The interview takes approximately 30 minutes, and there are no "right" or "wrong" answers, all input is useful to us. If there are any questions you don't quite understand or if you don't know what to answer, that's fine. It just means we need to make the questions a little better.

Since this is a telephone interview, there is a chance of misunderstandings. Since I don't see you, I can for example think that you have finished answering and I move on to the next question, while you are just having a pause to think. So, if that kind of misunderstanding happens and I don't catch it, please let me know.

As mentioned in the consent letter, I am going to record the interview, and this because I want to be sure that I catch what is being said. We take responsibility for ensuring that all information you share with us will be kept safe and secure. Before we get started, do you have any questions?
Ok, I'll start recording the call then. I will now put you on loudspeaker, but you should know that I'm the only person in the room. That's to get the sound on the tape recorder.

[Put on audio recorder]

Map the users' knowledge and experiences with digital treatment

The first thing I'm wondering is
Have you ever tried apps or other technology to get help with an eating disorder or other difficulties?

<u>Not</u> tried digital treatment
Have you heard anything about digital treatments?
If YES: What have you heard?

Tried digital treatment
How do you think it worked?
What was positive/negative [the adjective the participant uses]?
Was there anything that was negative/positive, possibly what?

Map the users' needs related to digital treatment

When we are going to develop an app or similar on mobile, PC or in VR, we need input on which topics we should focus on.

What do you think a digital treatment for eating disorders should contain?

Could you say a little about why you think we should focus on____________?

Is there anything else you think a digital treatment should contain?

I have some topics I want to review to see if you think they should be part of a digital treatment. Do you think that any of the following topics will be important to focus on:

Knowledge of eating disorders
If YES: Could you say a little about why you think we should focus on knowledge about eating disorders?

Low self-esteem
Perfectionism
If YES: Could you say a little about why you think we should focus on_______________?

Concern about food, calories and eating
Concern for appearance and figure
Concern about weight
If YES: Could you say a little about why you think we should focus on_______________?

Difficulties with friends
Difficulties in the family
Difficulties at school
If YES: Could you say a little about why you think we should focus on_______________?

Anxiety and anxiety symptoms
Sadness and depressive symptoms
Dealing with difficult emotions
Feeling of losing control
If YES: Could you say a little about why you think we should focus on_______________?

The young person answers YES to <u>MORE</u> than 3 topics:
If you were to choose 3 of these topics that you think a digital treatment for young people with eating disorders MUST contain, which 3 topics would they be?
How would you rank these topics, from 1–3?

The young person answers YES to <u>ONLY</u> 3 topics:
How would you rank these topics, from 1–3?

Is there any other topic that you think a digital treatment for eating disorders should contain?

Could you say a little about why you think we should focus on _____________________?

Do you think digital treatment can make everyday life easier for young people with eating disorders?
If YES: In what way?
If NO: Could you say a little about why not?

Many people who have an eating disorder may find it difficult to seek help and accept treatment. Do you think a digital treatment can help increase motivation for treatment?

If YES: In what way?
If NO: Could you say a little about why not?

Other things that we might want to think about when creating a digital treatment for young people with eating disorders?

Is there anything a digital treatment for young people with eating disorders should not contain?

Map users' interest in digital treatment

The next question contains some text. I therefore want to send you a picture via SMS that contains the text I am going to read out loud. Is that okay? [send photo by sms] *The text contains four examples of what a digital treatment for eating disorders might look like. It is not that we think digital treatment should be one of these options, they are only examples.*

A digital treatment for eating disorders can, for example, be:

A) An app where you can learn about the eating disorder, keep a food diary, enter homework, record weight, etc.

B) An app where you can chat with the therapist.

C) An app where one can do behavioral experiments in virtual reality (VR), e.g. testing different assumptions when shopping at the store, eating lunch at school, eating with the family, etc.

D) A computer game with different levels where you expose yourself to anxiety-inducing situations related to conditions of the eating disorder, e.g. perfectionism, low self-esteem.

If you were to rank these options from 1-4 according to how relevant you think the treatment may be for young people with eating disorders, in what order would you rank them?

Could you say a little about why you gave __A-B-C-D___ **a top rating?**

Could you say a little about why you ranked _A-B-C-D__ **at the bottom?**

Now that we are nearing the end; Is there something I forgot to ask you?

Is there anything you want to ask me?

Thank you very much for your quality input. This will really help us develop further!
