# Supplementary material for: Adolescents’ perspectives on a novel digital treatment targeting eating disorders: a qualitative study
Source: BMC Psychiatry. 2024 Jun 5;24:423. doi: 10.1186/s12888-024-05866-1 (PMC11155031; doi:10.1186/s12888-024-05866-1)
Supplement: Supplementary file 2 — Supplementary Material 2: Additional file 2 (docx). Title of data: Consolidated criteria for reporting qualitative research (COREQ). Description of data: Reporting guidance for qualitative research [file 12888_2024_5866_MOESM2_ESM.pdf]

Consolidated criteria for reporting qualitative research (COREQ):

No.	Item	Guide questions/ descriptions	Responses
**Domain 1: Research team and reflexivity**			
1.	Interviewer/facilitator	Which author/s conducted the interview or focus group?	GH: 10 interviews, SEAD: 3 interviews, SEAD: 3 interviews.
2.	Credentials	What were the researcher's credentials?	GH: PhD-candidate, SEAD: clinical psychology student, SFD: clinical psychology students, EMSP: PhD, IBE: MD PhD, TN: PhD.
3.	Occupation	What was their occupation at the time of the study?	GH: Clinical social worker/ PhD-candidate, SEAD: Clinical psychology student, SFD: Clinical psychology student, EMSP: Clinical psychologist, IBE: Medical doctor/Professor, TN: Clinical psychologist/ Professor.
4.	Gender	Was the researcher male or female?	Female
5.	Experience and training	What experience or training did the researcher have?	The senior researchers (EMSP, IBE, and TN) and the PhD candidate all have previous experience in qualitative research
6.	Relationship established	Was a relationship established prior to study commencement?	No
7.	Participant knowledge of the interviewer	What did the participants know about the researcher?	The participants were informed about the reasons for the project (developing a novel digital treatment)
8.	Interviewer characteristics	What characteristics were reported about the interviewer/facilitator?	No characteristics were reported
**Domain 2: study design**			
9.	Methodological orientation and Theory	What methodological orientation was stated to underpin the study?	We used reflexive thematic analysis to guide the design and analysis of the study.
10.	Sampling	How were participants selected?	We used convenience sampling method.
11.	Method of approach	How were participants approached?	Participants were approached by posters, social media, and telephone.
12.	Sample size	How many participants were in the study?	16
13.	Non-participation	How many people refused to participate or dropped out? Reasons?	4 participant dropped-out before the interviews.

			Reasons was not specifically recorded.
14.	Setting of data collection	Where was the data collected?	Telephone
15.	Presence of non-participants	Was anyone else present besides the participants and researchers?	No
16.	Description of sample	What are the important characteristics of the sample?	Mean age was 17.4 years (SD=1.01, range 16-19), all participants were female.
17.	Interview guide	Were questions, prompts, guides provided by the authors? Was it pilot tested?	The interview guide was semi structured with closed and open-ended questions. The interview guide was not piloted, but a panel with expertise was assembled to assess the effectiveness of the interview questions.
18.	Repeat interviews	Were repeat interviews carried out? If yes, how many?	None were repeated
19.	Audio/visual recording	Did the research use audio or visual recording to collect the data?	Interviews were audio-recorded
20.	Field notes	Were field notes made during and/or after the interview or focus group?	The interviews kept reflexive research journals.
21.	Duration	What was the duration of the interviews?	The duration was 25-46 minutes. Mean time was 34 minutes
22.	Data saturation	Was data saturation discussed?	The authors discussed saturation during the analysis process.
23.	Transcripts returned	Were transcripts returned to participants for comment and/or correction?	No
**Domain 3: analysis and findings**			
24.	Number of data coders	How many data coders coded the data?	All transcripts were coded by the first author. See paragraph "Reflexivity".
25.	Description of the coding tree	Did authors provide a description of the coding tree?	See paragraph "Data analysis" and Table 1
26.	Derivation of themes	Were themes identified in advance or derived from the data?	Themes were derived from the data.
27.	Software	What software, if applicable, was used to manage the data?	The NVivo software was used to manage the data.
28.	Participant checking	Did participants provide feedback on the findings?	No
29.	Quotations presented	Were participant quotations presented to illustrate the themes / findings? Was each quotation identified?	Several quotations are presented in the manuscript. Each participant was given a

			number which was used to identify the quotes.
30.	Data and findings consistent	Was there consistency between the data presented and the findings?	Yes
31.	Clarity of major themes	Were major themes clearly presented in the findings?	Yes
32.	Clarity of minor themes	Is there a description of diverse cases or discussion of minor themes?	Yes
